# Synthetic Strategies Toward Nitrogen-Rich Energetic Compounds *Via* the Reaction Characteristics of Cyanofurazan/Furoxan

**DOI:** 10.3389/fchem.2022.871684

**Published:** 2022-03-17

**Authors:** Luoluo Wang, Lianjie Zhai, Weiqing She, Minchang Wang, Junlin Zhang, Bozhou Wang

**Affiliations:** State Key Laboratory of Fluorine & Nitrogen Chemicals, Xi’an Modern Chemistry Research Institute, Xi’an, China

**Keywords:** nitrogen-rich energetic compounds, cyanofurazan/furoxan, synthesis, reaction mechanism, energetic property

## Abstract

The structural units of amino-/cyano-substituted furazans and furoxans played significant roles in the synthesis of nitrogen-rich energetic compounds. This account focused on the synthetic strategies toward nitrogen-rich energetic compounds through the transformations based on cyanofurazan/furoxan structures, including 3-amino-4-cyanofurazan, 4-amino-3-cyano furoxan, 3,4-dicyanofurazan, and 3,4-dicyanofuroxan. The synthetic strategies toward seven kinds of nitrogen-rich energetic compounds, such as azo (azoxy)-bridged, ether-bridged, methylene-bridged, hybrid furazan/furoxan-tetrazole–based, tandem furoxan–based, hybrid furazan-isofurazan–based, hybrid furoxan-isoxazole–based and fused framework–based energetic compounds were fully reviewed, with the corresponding reaction mechanisms toward the nitrogen-rich aromatic frameworks and examples of using the frameworks to create high energetic substances highlighted and discussed. The energetic properties of typical nitrogen-rich energetic compounds had also been compared and summarized.

## Introduction

Nitrogen-rich aromatic structures are the most essential frameworks for the constructions of nitrogen-rich energetic compounds, which constitute the core component shared by some most powerful explosives and propellants (Singh et al., 2007; Viswanath et al., 2018; Gao et al., 2020; Zhou et al., 2021). Unlike traditional energetic compounds, nitrogen-rich energetic compounds generate environmentally friendly molecular nitrogen as the major end-product of propulsion or explosion and have been the focus of research into energetic materials worldwide (Yin and Shreeve, 2017; Klapötke, 2021). During the past decades, heterocyclic five-membered rings of furazan, furoxan, isofurazan (1,2,4-oxadiazole and 1,3,4-oxadiazole), tetrazole, isoxazole, and their fused derivatives (such as pyridofurazan, triazolofurazan, pyridazinofuroxan etc.) are the most popular choice in the creation of new nitrogen-rich energetic compounds (Tang et al., 2016a; Zhang et al., 2017; Huang et al., 2020; Voronin et al., 2020; Yan et al., 2020; Dalinger et al., 2021; Larin et al., 2021). From the structural point of view, the frameworks based on the combinations of these heterocyclic five-membered rings, especially the ones with furazan, furoxan, tetrazole, or isofurazan units, can achieve more compact structures and higher enthalpy of positive formations, leading to corresponding nitrogen-rich energetic compounds with excellent detonation performances (Jaidann et al., 2010; Swain et al., 2010; Suntsova and Dorofeeva, 2017).

The synthetic pathways to frameworks, or more often their functionalized derivatives, suitable for the various required nitrogen-rich energetic compounds usually depend on key intermediates with functional groups that enable diverse and desired transformations (Agrawal and Hodgson, 2007). Compared with other nitrogen-rich heterocycles, most furazan- and furoxan-based energetic heterocycles demonstrate superior energetic properties and better modifiability, making them as a most successful class of structures for the design and synthesis of energetic materials. From a practical standpoint, cyanofurazan/furoxan has been regarded as one of the most important energetic intermediates for the synthesis of nitrogen-rich energetic compounds comprising furazan and furoxan structures, and these key cyanofurazan/furoxan structures mainly include 3-amino-4-cyanofurazan, 4-amino-3-cyanofuroxan, 3,4-dicyanofurazan, and 3,4-dicyanofuroxan. From a synthetic standpoint, cyano groups in cyanofurazan/furoxan structures enjoy a high degree of transformational diversity and can be further transformed into other heterocyclic five-membered rings such as furoxan, isofurazan, tetrazole, and isoxazole, leading to a series of linear or fused frameworks of nitrogen-rich energetic compounds (Qu et al., 2016; Pagoria et al., 2017; Wu et al., 2017; Zhai et al., 2019a; Johnson et al., 2020). The cyano groups could also be easily turned to highly powerful explosophoric groups of dinitromethyl, trinitromethyl, and fluorodinitromethyl groups (Gu et al., 2018). Moreover, the additional amino groups in cyanofurazan/furoxan structures provide perfect precursors for the synthesis of azo/azoxy moieties (Luo et al., 2010; Li et al., 2014).

**FIGURE 1:**
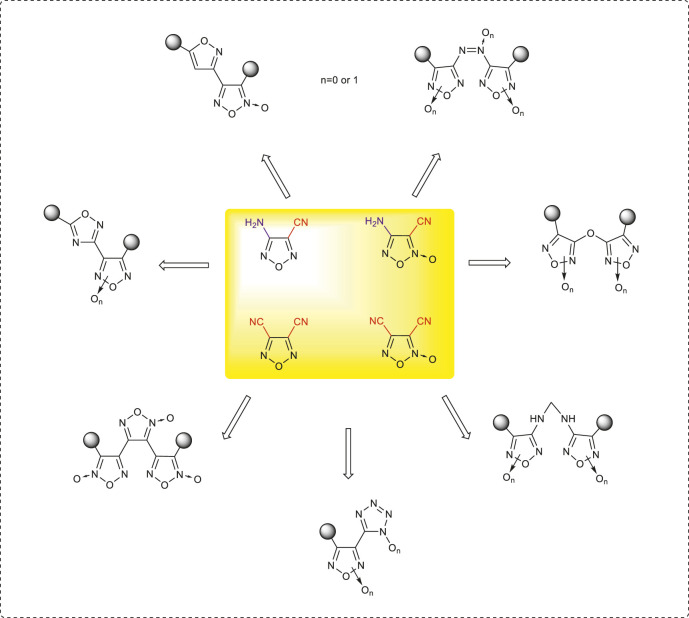
Synthetic strategies toward nitrogen-rich frameworks based on cyanofurazans/furoxans.

The first part of the account focuses on the synthetic methods of 3-amino-4-cyanofurazan, 4-amino-3-cyanofuroxan, 3,4-dicyanofurazan, and 3,4-dicyanofuroxan and the abovementioned key intermediate structures and building blocks. The detailed reaction mechanisms for their preparations were also discussed in this part. It then outlines the recent successful manipulation of these intermediate structures/building blocks for the design and synthesis of corresponding nitrogen-rich energetic compounds. Based on the active cyano and amino groups, the introductions of azo/azoxy moieties, the formations of fused energetic heterocycles, the constructions of liner structures coupled *via* C–C or C–O bonds by bringing in additional heterocycles (including tetrazole, furazan, furoxan, isoxazole, and isofurazan), and the physicochemical properties and detonation performances of typical energetic compounds were fully reviewed (Figure 1). New structural design concepts and promising synthetic strategies related were also discussed.

## Synthesis of Cyano-/Amino-Substituted Furazan and Furoxan Intermediates

The synthetic intermediates, including 3-amino-4-cyanofurazan, 4-amino-3-cyanofuroxan, 3,4-dicyanofurazan, and 3,4-dicyanofuroxan, are the basis and key building blocks for the synthetic work toward numerous nitrogen-rich energetic compounds. In general, the synthesis of these cyano-/amino-substituted furazans and furoxans involves the preparations of specific cyclized precursors and complex functional group transformation mechanism, which also provides important references for the design and synthesis of other cyano-/amino-substituted heterocycles.

### Synthesis of 3-Amino-4-cyanofurazan and 4-Amino-3-cyanofuroxan

**SCHEME 1:**
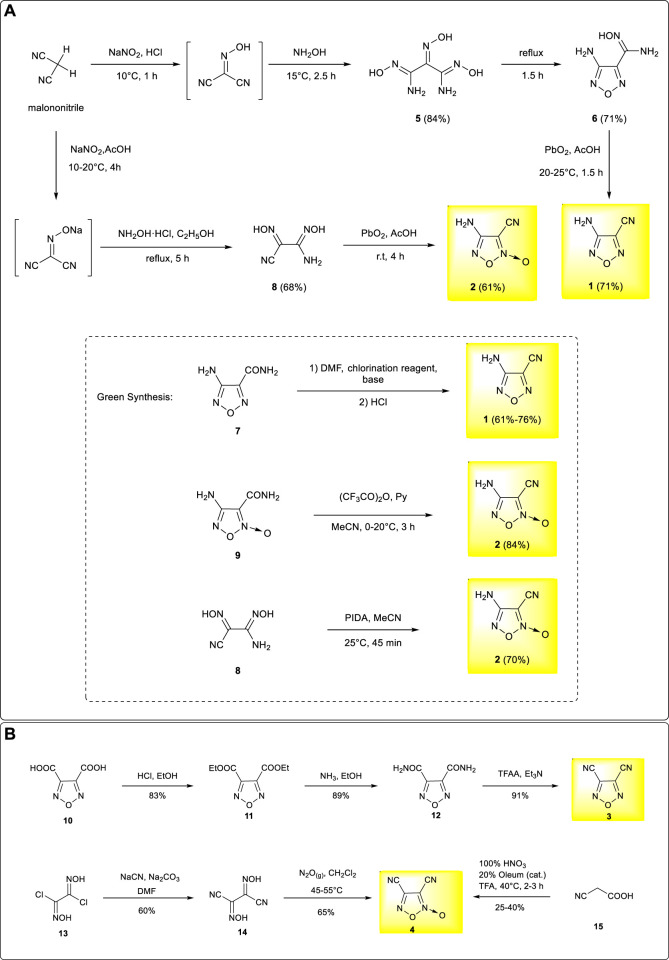
**(A)** Synthesis of 3-amino-4-cyanofurazan and 4-amino-3-cyanofuroxan; **(B)** Synthesis of 3,4-dicyanofurazan and 3,4-dicyanofuroxan.

Based on malononitrile, an active methylene compound as the starting material, the core furazan framework was formed through nitrosation and oximation reactions followed by dehydration and cyclization. The treatment of the hydroxylamine moiety with lead dioxide in acetic acid solution led to the formation of 3-amino-4-cyanofurazan **1** with a yield of 71% as the major product (Fan et al., 2008; Pagoria et al., 2017). The heavy metal reagents participate in the final elimination process and generate intensive heavy metal residues in the wastewater, causing serious pollution. To avoid the use of heavy metal reagents, an alternative synthetic method of 3-amino-4-cyanofurazan was developed based on 4-aminofurazan-3-formamide **7**, which was obtained from N,N-dimethylformamide, a chlorination reagent, and an organic base. 3-amino-4-cyanofurazan was then obtained through a dehydration process (Fershtat et al., 2015). This method was much more environment-friendly and, therefore, more practical for scalable synthesis, providing fast access to large quantities of the target material for industrial production. Similar to the synthetic methods of 3-amino-4-cyanofuroxan, the synthesis of 4-amino-3-cyanofuroxan could also be achieved from malononitrile, but the synthesis pathway is more straightforward. After similar nitrosation and oximation reactions, the treatment of the dioxime moiety with lead dioxide gave the desired 4-amino-3-cyanofuroxan in 42% overall yields. (Luo et al., 2010). The alternative “green” synthesis of 3-amino-4-cyanofuroxan was also developed by dehydration of 4-aminofuroxan-3-formamide **9** under the medium of (CF_3_CO)_2_O/Py with a high reaction yield of 84% (Fershtat et al., 2015). More recently, another green and mild access to 3-amino-4-cyanofuroxan was further achieved through the oxidation of **8** by (diacetoxyiodo)benzene (PIDA) (Zhang et al., 2022). (Scheme 1A)

### Synthesis of 3,4-Dicyanofurazan and 3,4-Dicyanofuroxan

Compared with 3-amino-4-cyano–substituted furazan and furoxan, 3,4-dicyano–substituted furazan and furoxan are better substrates for the design and synthesis of symmetrical energetic structures. 3,4-dicyanofurazan was prepared based on furazan-3,4-dicarboxylic acid, a commercially available chemical. The esterification and amination of the carboxyl groups led to furazan-3,4-dicarboxamide **12**, and 3,4-dicyanofurazan **3** was obtained under the treatment of trifluoroacetic anhydride (TFAA) as a dehydrating agent with a yield approximated to 100% (Beyerle, 1983; Godovikova et al., 2009; Guo et al., 2018). In contrast, the preparation of 3,4-dicyanofuroxan was based on the commercially available chemical of dichloroglyoxime. The cyanation followed by a cyclization process yielded 3,4-dicyanofuroxan **4** in moderate yield (Grundmann et al., 1975). 3,4-dicyanofuroxan **4** could also be prepared through the nitration of cyanoacetic acid in trifluoroacetic acid medium, but with a poor yield (Parker et al., 1962). By using the dichloromethane solvent instead of trifluoroacetic acid (TFA), a much higher yield was achieved (72%), and the operational safety was also improved (Johnson et al., 2019). (Scheme 1B)

## Synthesis of Nitrogen-Rich Energetic Compounds

### Azo (azoxy)-Bridged Energetic Compounds

**SCHEME 2:**
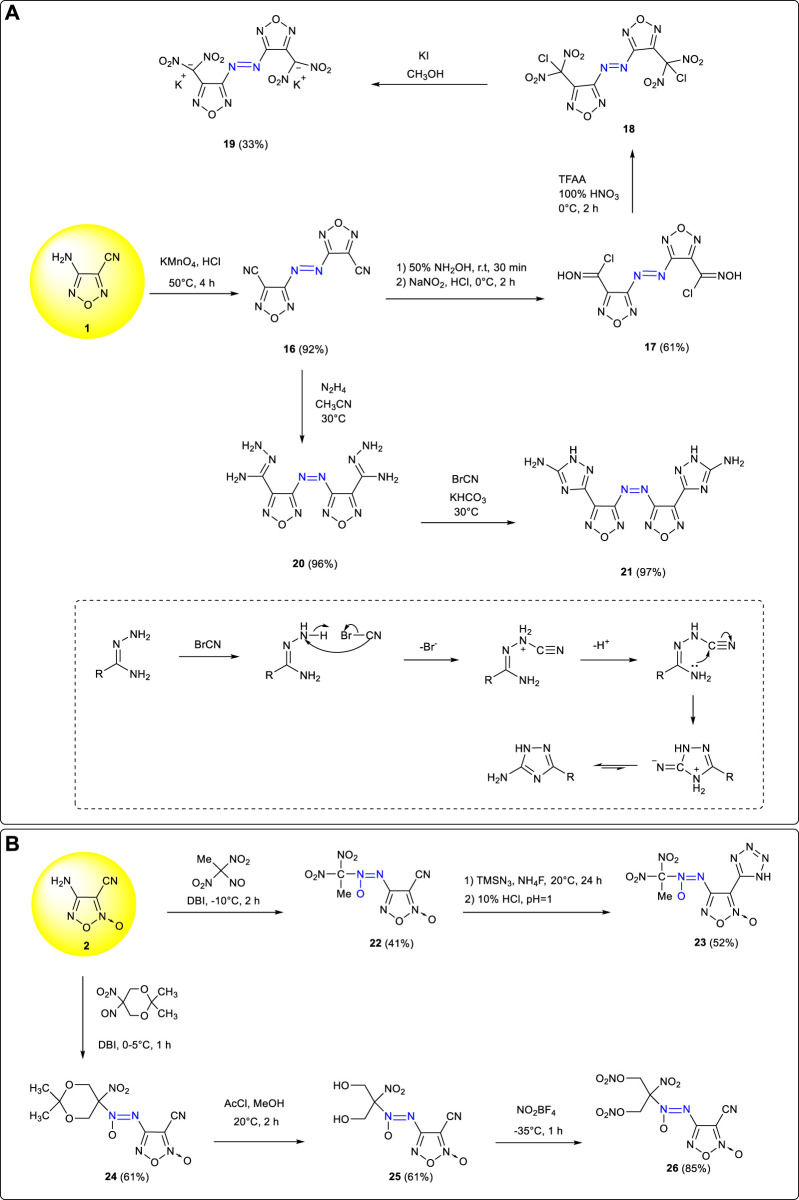
**(A)** Synthesis of **19 and 21** and the mechanism of cyclization process *via* hydrazide and BrCN; **(B)** Synthesis of **23** and **26** by oxidative coupling.

Azo and azoxy groups, which require the formation of two covalent bonds, are a bridge between two identical or different frameworks. Because of the inherent greater endothermicity of the N=N bond, the constructions of azo and azoxy groups are very useful for creating energetic compounds with a high enthalpy of formation and contribute markedly to the overall energetic performance. Compared with monocyclic compounds, azo-bridged bicyclic compounds had more reaction activity sites and could be modified by more substituent groups so as to enrich the diversity of energetic materials (Qu and Babailov, 2018; Kozak et al., 2008; Türker., 2016; Chavez et al., 2000; Liu et al., 2016; Sheremetev et al., 1998). Based on the reaction characteristics of the amino group in 3-amino-4-cyanofurazan **1**, the corresponding azo compound 3,3'- dicyano-4,4'-azofurazan **16** (ρ: 1.62 g cm^−3^, T_d_: 234°C, D: 7,640 m s^−1^, P: 21.8 GPa) was obtained by the oxidation of potassium permanganate in acidic medium (Fan et al., 2008). Due to the high reaction activity of the cyano group, energetic groups such as geminal-dinitro (-CH(NO_2_)_2_) and triazole ring could be introduced to significantly improve the properties of **16** derivatives. Using **16** as the starting material, a three-dimensional energetic metal organic skeleton potassium 4,4'-bis (dinitromethyl)-3,3'-azofurazanate **19** was obtained by Tang et al. through addition, diazotization, nitration, and reduction reactions (Tang et al., 2016b). It was a promising green explosive with good thermal stability and detonation performances (T_d_: 229°C, D: 8,138 m s^−1^, P: 30.1 GPa), an impact sensitivity of 2 J, and a friction sensitivity of 20 N to external stimuli. In addition, triazole energetic compound **21** was synthesized by Qu et al. from **16** through hydrazine addition and cyclization (Qu et al., 2016). (Scheme 2A) The cyclization mechanism is also shown in Scheme 2A: Under alkaline conditions, HBr was eliminated because BrCN attacked the N atom on the hydrazino group; then the lone pair electrons of nitrogen on the amino group attacked the C atom on the cyano group (-CN) to form a C–N bond, and then through proton transfer, a triazole ring structure could be formed. Compared with **16**, the thermal stability and detonation performances of **21** were improved to a certain extent (T_d_: 309°C, D: 8,458 m s^−1^, P: 26.2 GPa). In order to further improve the detonation performance of energetic compounds, the method of H_2_O_2_/H_2_SO_4_ oxidation or NaNO_2_/H_2_SO_4_ diazotization–substitution (Zhang et al., 2020) could be used to convert amino groups (-NH_2_) into nitro groups (-NO_2_).

Based on the oxidative couplings between primary amines and nitroso compounds, a series of azoxy energetic compounds were synthetized by Parakhin et al. using 4-amino-3-cyanofuroxan **2** as starting materials (Parakhin et al., 2017). The azoxy intermediate **22** obtained by reacting with 1,1-dinitro-1-nitroethane further reacted with TMSN_3_ by [3 + 2] cycloaddition, and tetrazolyl was successfully introduced to obtain a new energetic compound **23**. Moreover, the energetic intermediate **25** was obtained from **2** and 2,2-dimethyl-5-nitro-5-nitroso-1,3-dioxane by oxidative coupling using DBI (dibromocyanuric acid) and then hydrolysis. Using **25** as the intermediate, compound **26** could be obtained by the nitration of NO_2_BF_4_ introducing into nitrate groups. The dinitromethyl structure could also be obtained through bromination, reduction, and nitration from **26** so as to further improve the energy and density of target energetic compounds (Zhang et al., 2016). (Scheme 2B)

### Ether-Bridged Energetic Compounds

**SCHEME 3:**
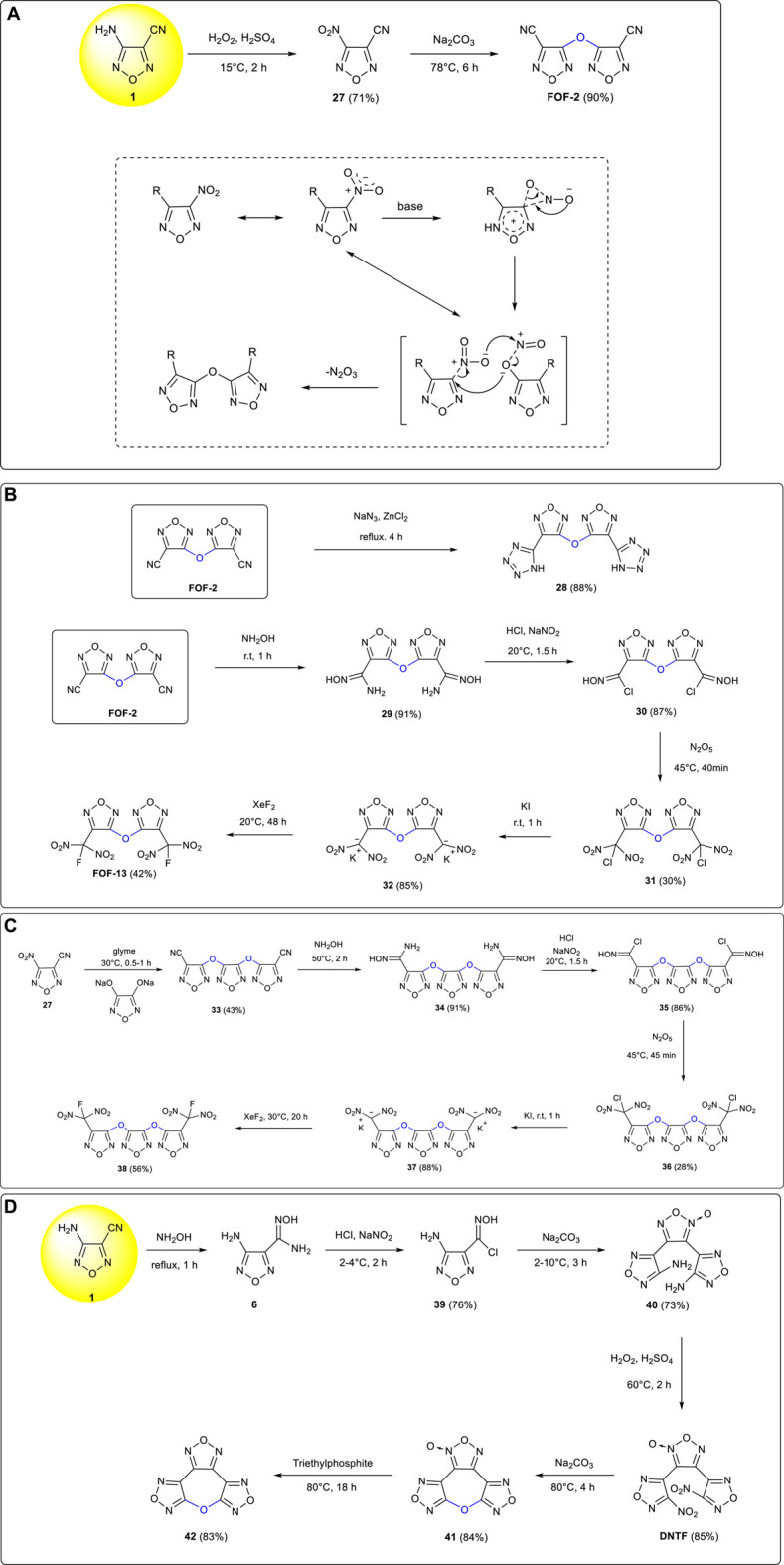
**(A)** Synthesis of **FOF-2**; **(B)** synthesis of **28** and **FOF-13**; **(C)** synthesis of **37** and **38**; **(D)** synthesis of **41** and **42**.

Bridging multiple furazan through ether bonds can significantly enhance the density level, improve oxygen balance, and increase flexibility of the aromatic molecules. Most furazanyl ether compounds exhibit high energy density, high standard enthalpy of formation, high nitrogen content, low melting point and strong plasticity, making them ideal candidates as energetic plasticizers or oxidant components in low signal characteristic propellant (Wang et al., 2012).,

Starting from 3-amino-4-cyanofurazan **1**, 3-nitro-4-cyanofurazan **27** was provided under Caro’s acid oxidation conditions (Youssif, 2001). A novel intermolecular etherification was carried out under alkaline conditions with the oxygen bridged compound 3,3-dicyanodifurazan ether **FOF-2** obtained in good yield (Fan et al., 2009). (Scheme 3A) With an impact sensitivity of 0%, a friction sensitivity of 0%, and an H_50_ value greater than 125.9 cm, **FOF-2** was regarded as a good candidate of an insensitive high-energy plasticizer and used as an important starting material for the synthesis of other furazan energetic compounds.

3,3’-Bis (tetrazol-5-yl) difurazanyl ether **28** (ρ: 1.87 g cm^−3^, D: 8,720 m s^−1^, P: 33.41 GPa) was synthetized by Li et al. from [3 + 2] cycloaddition between **FOF-2** and sodium azide in 88% yield. The contained intensive hydrogen bond interactions increased the thermal stability of the target compound substantially (Li et al., 2012). 3.3'-Bis (fluorodinitromethyl) difurazanyl ether (**FOF-13**) was prepared by Wang et al. from FOF-2 through the five-step reactions of cyano group addition, diazotization, N_2_O_5_ nitration, KI reduction, and XeF_2_ fluorination (Wang et al., 2014). (Scheme 3B) **FOF-13** is regarded as a potential energetic plasticizer due to its high density of 1.97 g cm^−3^, good thermal stability (no obvious thermal decomposition below 270°C), and excellent compatibility with most other components of mixed explosives and solid rocket propellants.

3-Nitro-4-cyanofurazan **27**, a substance obtained by the oxidation of 3-amino-4-cyanofurazan **1**, was etherified with 3,4-dihydroxyfurazan sodium salt and yielded an oxygen-bridged trifurazan ether intermediate of **33** with a yield of 89% (Sheremetev et al., 1998). A new green primary explosive 3,4-bis(3-dinitromethylfurazan-4-oxy) furazan **37** (ρ: 2.09 g cm^−3^, D: 8,431 m s^−1^) was synthesized by Zhai et al. *via* the four-step reactions of addition, diazotization, nitration and reduction. Compared with the common applied primary explosive of lead azide; **37** was a nontoxic compound and showed stronger initiation ability (Zhai et al., 2015a). In the following year, 3,4-bis(3-fluorodinitromethylfurazan-4-oxy) furazan **38**, which showed the characteristics of good thermal stability (T_d_: 197.8°C), high density (1.88 g cm^−3^), low melting point (50°C), and high energy level (D: 8,644.5 m s^−1^, P: 34.0 GPa), was also obtained by Zhai et al. from further fluorination of **37** (Scheme 3C). The newly developed compound could be used as an energetic plasticizer with excellent comprehensive properties (Zhai et al., 2016a).

3,4-Bis (4'-nitrofurazan-3'-yl) furoxan (**DNTF**) was synthesized from 3-amino-4-cyanofurazan **1** *via* the four-step reactions of cyano addition, diazotization–chlorination, intermolecular cyclization, and oxidation (Zhou et al., 2011). Bifurazano[3,4-b:3',4'-f] furoxano[3″,4″-d] oxacyclohetpatriene **41** (ρ: 1.866 g cm^−3^, m.p.: 92°C, D: 8,256 m s^−1^, H_50_: 57.5 cm) was obtained by Zhou et al. *via* intramolecular etherification of **DNTF** under the basic conditions of Na_2_CO_3_ in acetonitrile solution (Zhou et al., 2012). Trifurazanoxyheterocycloheptene **42** (ρ: 1.935 g cm^−3^, m.p.: 76.5°C, D: 8,646 m s^−1^, H_50_: 72.4 cm) was further developed by Wang et al. from the triethylphosphite reduction of **41** with a yield of 83% (Wang et al., 2020). (Scheme 3D) These two cyclic furazan ether energetic compounds exhibited the characteristics of low melting point, low sensitivity, and high energy density and were potential liquid carrier explosive components in melting and casting explosives.

### Methylene-Bridged Energetic Compound

**SCHEME 4:**
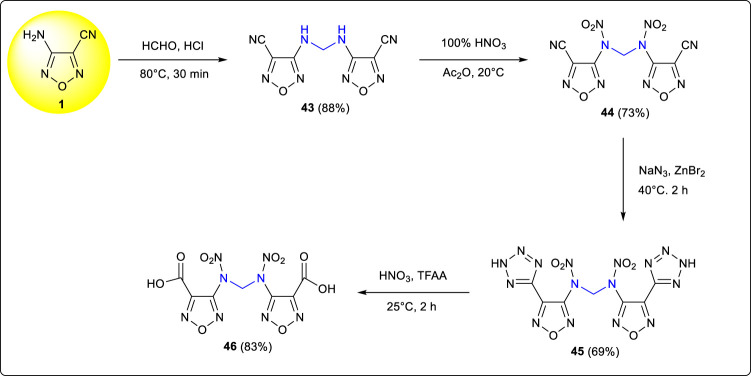
Synthesis of 45 and 46.

Methylene-bridged compound **43** was developed by Sun et al. from the condensation reaction of 3-amino-4-cyanofurazan and formaldehyde (Sun et al., 2017). *N,N’*-methylenebis(n-(4-(2H-tetrazol-5-yl) 1,2,5-oxadiazol-3-yl) nitramide) **45**, which showed excellent energy performances (D: 9,043 m s^−1^, P: 35.6 GPa) and acceptable sensitivities (IS:16 J, FS:180 N), was obtained by the nitration and addition reactions. It was noteworthy that the tetrazole group was converted to a carboxyl group when **45** was nitrated with the mixed 100% nitric acid and trifluoroacetic anhydride; the rarely reported method provided a new strategy for the preparations of heterocyclic carboxylic acids (Scheme 4).

### Hybrid Furazan/Furoxan-Tetrazole–Based Energetic Compounds

Tetrazoles have high nitrogen content and high positive enthalpy of formation and are, therefore, very suitable as building blocks for the development of energetic materials. The hybridization of tetrazoles and furazans/furoxans was an effective strategy to construct a series of energetic compounds with hybrid furazan/furoxan-tetrazole frameworks, which also greatly expand the research and applications of tetrazole-based energetic substances (Kumar et al., 2017).

**SCHEME 5:**
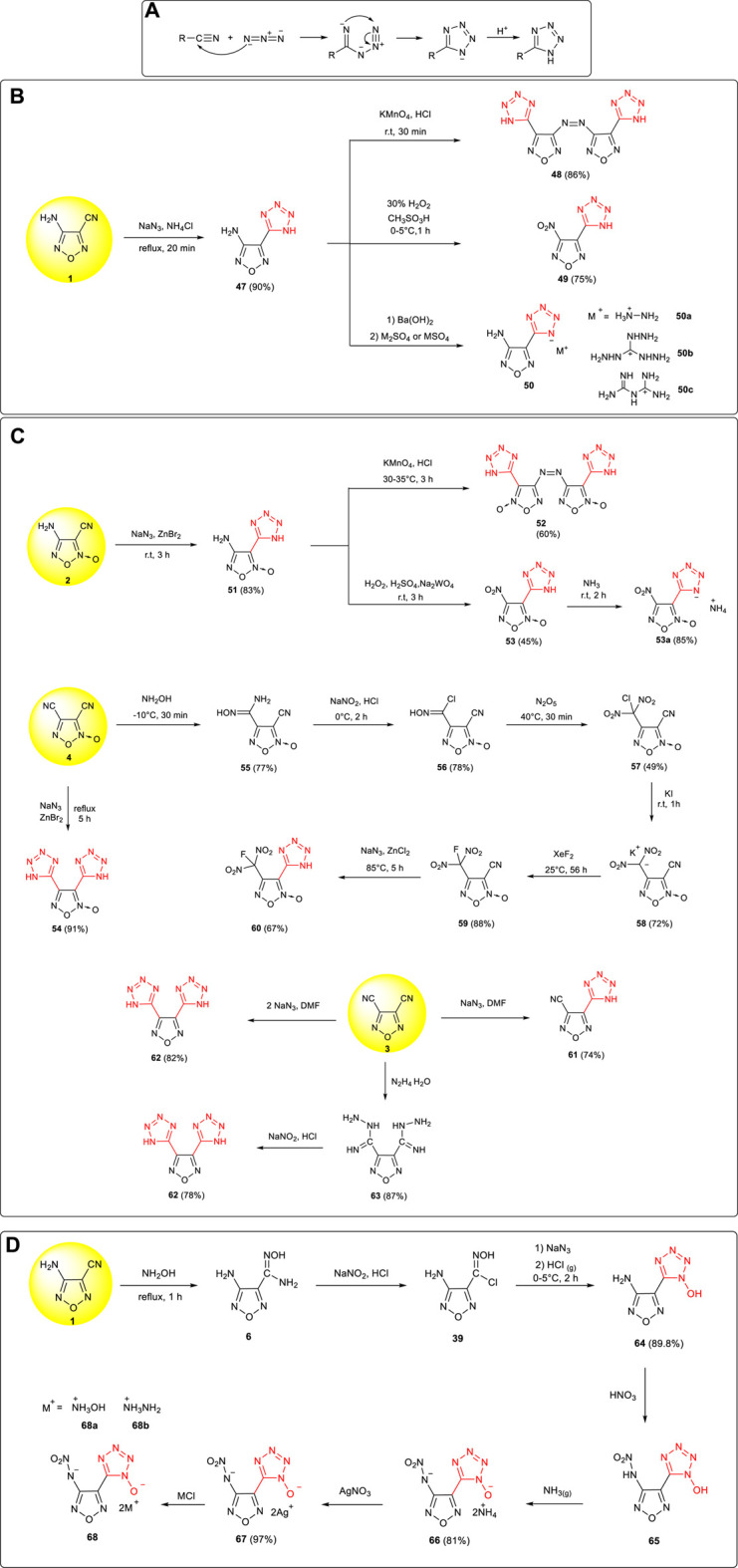
**(A)** Mechanism of 1,3-dipole cycloaddition reaction toward tetrazoles; **(B)** Synthesis of **48**, **49**, and **50** and its energetic ionic salts; **(C)** synthesis of **52**, **53**, **54**, **60, 61,** and **62**; **(D)** synthesis of **68** and its energetic ionic salts.

The cycloaddition reaction between cyano and azido moieties was one of the important methods in the synthesis and construction of tetrazoles, and the mechanism of the transformation is shown in Scheme 5A: under acidic conditions, tetrazole structure was formed from the 1,3-dipole cycloadditions between cyano and azido groups (Fershtat et al., 2015). Using cyanofurazan (furoxan) as the starting material, a series of tetrazole energetic compounds with excellent properties have been prepared.

3-Amino-4-(tetrazol-5-yl) furazan **47** was synthetized by Wang et al. *via* cycloaddition from 3-amino-4-cyanofurazan **1** with a yield of 90% (Wang et al., 2011). Based on the activity of the amino group and tetrazole group in **47**, a series of new energetic compounds were successfully obtained. 3,3-bis(1H-tetrazol-5-yl)-4,4-azofurazan **48** (ρ: 1.69 g cm^−3^, D: 8,477 m s^−1^) was prepared by the oxidation of **47** by potassium permanganate under acidic conditions, and 3-nitro-4-(1H-tetrazol-5-yl) furazan **49** (ρ:1.67 g cm^−3^, D:8,257 m s^−1^) was prepared by the oxidation of the amino group (Gao et al., 2013). In addition, three nitrogen-rich energetic salts were obtained by using the acidity of tetrazole in **47**, which could be potential energetic components in the propellant (Scheme 5B)

Based on the similar transformations, 3,3’-bis (1H-tetrazol-5-yl)-4,4’-azofuroxan **52**, 4-nitro-3-(1H-tetrazol-5-yl) furoxan **53,** and its energetic salt were prepared from 4-amino-3-cyanofuroxan **2** (Zhai et al., 2018; Liang et al., 2013). The detonation performance for the ammonium salt of **53** (ρ: 1.84 g cm^−3^, D: 8,919 m s^−1^, P: 36.2 GPa) was close to that of RDX. As shown in Scheme 5C, using 3,4-dicyanofuroxan **4** as the starting material, two tetrazole frameworks were introduced to obtain 3,4-bis (1H-tetrazol-5-yl) furoxan **54** (ρ: 1.62 g cm^−3^, D: 7,778 m s^−1^, P: 23.9 GPa) (Zhai et al., 2015b). From 3,4-dicyanofurazan **3**, some tetrazolylfurazans could be obtained from nitrile precursors (Godovikova et al., 2009). After the convention of one cyano group to the fluorodinitromethyl group through cyanoaddition, diazotization, nitration, reduction, and fluorination, the other cyano group was then turned into a tetrazolyl moiety, leading to 3-(1H-tetrazol-5-yl)-4-fluorodinitromethylfuroxan **60** with excellent detonation properties (D: 8,923 m s^−1^, P: 36.3 GPa) and broad application prospects in solid rocket propellants and explosives (Zhai et al., 2020a).

The cycloadditions of chlorooxime and azide are widely applied in the synthesis of hydroxytetrazole compounds. Starting from 3-amino-4-cyanofurazan **1**, 5-(4-amino-1,2,5-oxadiazol-3-yl)-1-hydroxytetrazole **55** was prepared by Zhai et al. through NH_2_OH addition, diazotization–chlorination, NaN_3_ substitution, and cycloaddition reaction in an 89% yield (Zhai et al., 2016b). The nitroamino group in 5-(4-nitroamino-1,2,5-oxadiazol-3-yl)-1-hydroxytetrazole **65** was then introduced by Wei et al. through the nitration of **64**, based on which the corresponding hydroxylaminium salt **68a** and hydrazinium salt **68b** were further obtained (Wei et al., 2015a). The two energetic salts showed high density (1.84 g cm^−3^, 1.74 g cm^−3^), excellent detonation pressure (38.3 GPa, 32.2 GPa), and detonation velocity (9,323 m s^−1^, 9,094 m s^−1^), which were better than those of the high explosive of RDX and insensitive explosive of TATB (Scheme 5D)**.**

### Tandem Furoxan–Based Energetic Compounds

Tandem furoxans are significant energetic frameworks which have been widely applied in the development of HDEMs and achieved great success in recent years. Theoretical studies proved that replacing one nitro group with a furoxan moiety could increase the density of compounds by (0.06–0.08) g·m^−3^ and the corresponding detonation velocity by more than 300 m s^−1^ (Wang et al., 2021). Therefore, the design and synthesis of C–C bonded trifuroxan compounds was an effective method to obtain energetic compounds with outstanding energetic properties (Zhai, et al., 2019b).

**SCHEME 6:**
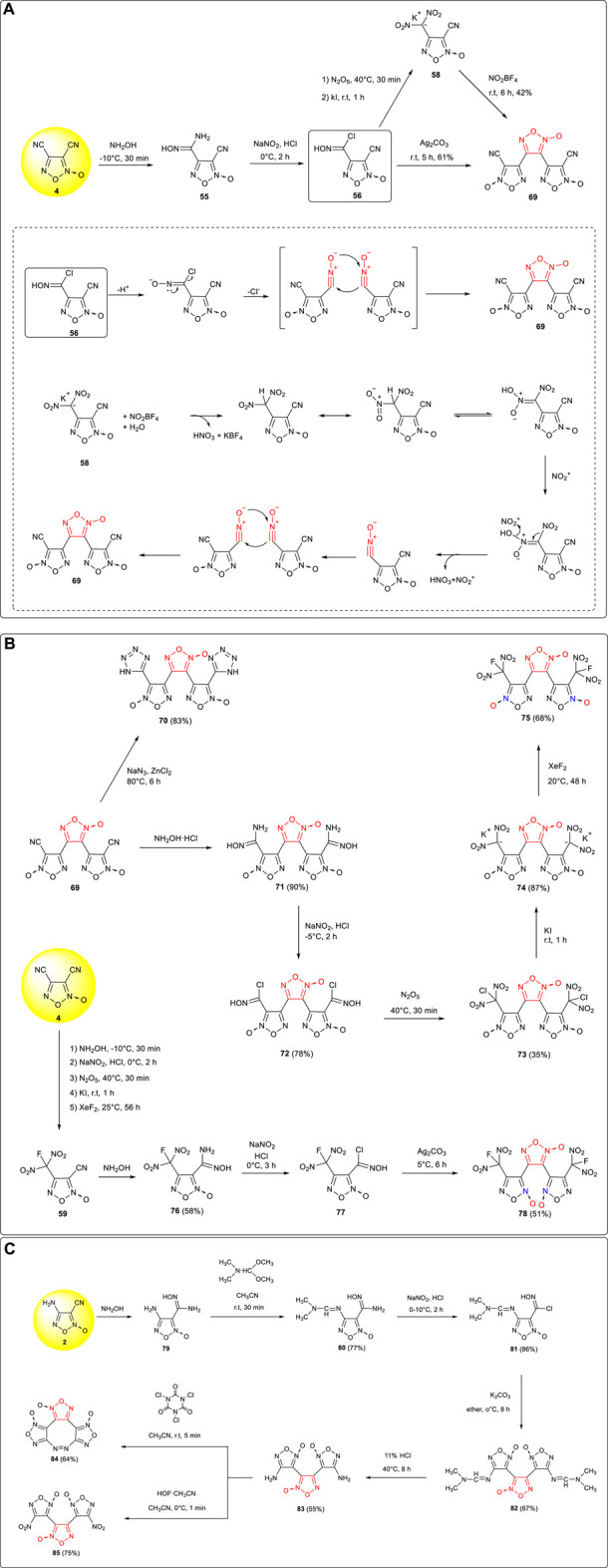
**(A)** Synthetic methods and reaction mechanism toward **69**; **(B)** synthesis of **70**, **75,** and **78**; **(C)** synthesis of **84** and **85**.

Chlorooxime intermediate **56** was prepared by Zhai et al. from 3,4-dicyanofuroxan **4** *via* the reaction process of cyano addition and diazotization, and a new type of energetic compound 3,4-bis (3-cyanofuroxan-4-yl) furoxan **69** was then obtained by two cyclization methods (Zhai et al., 2019c; Zhai et al., 2021): 1) under the condition of a weak base, HCl in **56** was removed and transformed into nitrile oxide structure and then furoxan was built by bimolecular dimerization–cyclization reaction for nitrile oxide; 2) the dinitromethyl potassium salt obtained by nitration of **56** was removed from a molecule of nitric acid and NO_2_^+^ under the action of NO_2_BF_4_ to form a nitrile oxide intermediate, and then the target compound was formed by the dimerization–cyclization of nitrile oxide (Scheme 6A).

Compound **69** was also an energetic intermediate with good expansibility, and using the reaction activity of the cyano group, tetrazolyl- (Zhai et al., 2020b) and fluorodinitromethyl-(Zhai et al., 2019c) substituted trifuroxan high-energy compounds could be obtained. Among them, 3,4-bis (3-tetrazolylfuroxan-4-yl) furoxan **70** had a very high heat of formation (1,290.8 kJ mol^−1^) and good detonation performances (D: 8,621 m s^−1^, P: 31.5 GPa), while 3,4-bis (3-fluorodinitromethylfuroxan-4-yl) furoxan **75** showed very excellent detonation performances (D: 9,509 m s^−1^, P: 42.6 GPa), which was close to those of CL-20. It is noteworthy that the synthesis sequence was changed under the same reaction conditions in each step; first, the cyano group in **4** was converted to the fluorodinitromethyl group; then, another cyano group was mainly dimerized and cyclized by a multistep reaction; last, the regional isomer 3,4-bis (4-fluorodinitromethylfuroxan-3-yl) furoxan **78** (D: 9,196 kJ mol^−1^, P: 38.8 GPa) was obtained in a 51% yield (Scheme 6B). Due to the synergistic effect between fluorodinitromethyl group and the N-oxidation bond of adjacent furoxan, the densities of the two isomers were significantly different (ρ (**75**): 2.00 g cm^−3^, ρ (**78**): 1.91 g cm^−3^). Therefore, this regiochemical modulation was expected to be an effective strategy to construct high-density energetic materials.

The amino group in **2** is too reactive and needs to be protected when NaNO_2_/HCl conditions are necessary. After the diazotization of aminooxime, dimerization and cyclization were carried out to provide the tandem furoxan framework. Deprotection reaction could be conducted under acidic conditions in the late stage, and the amino group given could be turned to nitro or azo moieties under oxidative conditions. Using this strategy, 3,4-bis(4’-aminofuroxano-3’) furoxan **83** was successfully synthesized by He et al. from 3-cyano-4-aminofuroxan (He et al., 2018). Under the oxidation of trichloroisocyanuric acid, an intramolecular azo bridge would be formed from **83**, and trifuroxan-fused 1,2-diazocine compound **84** (IS: 19 J, FS: 80 N, D: 9,417 m s^−1^, P: 39.2 GPa) was obtained in a 64% yield, which was expected to be a substitute for RDX and HMX. Under the condition of HOF-CH_3_CN, 3,4-bis (4’-nitrofuroxano-3’) furoxan **85** (ρ: 1.914 g cm^−3^, D: 9,503 m s^−1^, P: 40.8 GPa) was obtained in a 75% yield by the amino group being oxidized to the nitro group, and its high density, positive oxygen balance, and detonation properties were close to those of CL-20 (Scheme 6C).

### Hybrid Furazan–Isofurazan–Based Energetic Compounds

Although the formation enthalpy of isofurazan (1,2,4-oxadiazole and 1,3,4-oxadiazole) is lower than that of furazan, its stabilities toward external stimuli are much higher (Xue et al., 2019). Therefore, the introduction of isofurazans as desensitizing moieties to high-energy energetic compounds to form conjugate systems was an important strategy for the design and synthesis of low sensitivity and high-energy materials (Xue et al., 2020).

**SCHEME 7:**
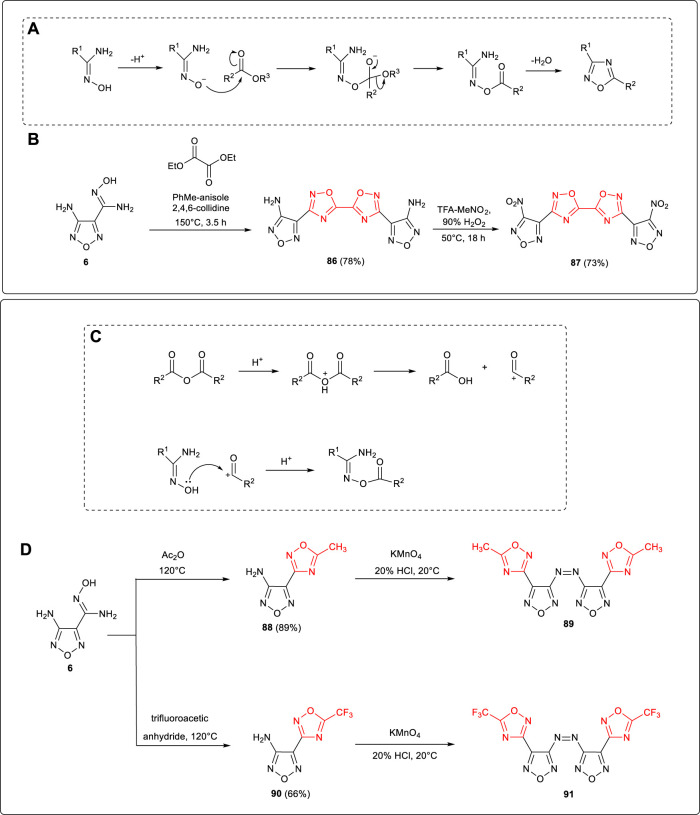
**(A)** Cyclization mechanism of amidoxime and ester; **(B)** synthesis of **87**; **(C)** O-acylation mechanism of amidoximes and anhydride *via* acid catalysis; **(D)** synthesis of **89** and **91**.

Amidoxime intermediate **6** obtained by cyano addition of 3-amino-4-cyanofurazan could be applied in the synthesis of various 1,2,4-oxadiazole energetic compounds with carboxylic acid derivatives and nitriles. From a mechanical point of view, 1,2,4-oxadiazole was synthetized from amidoxime and ester under alkali catalysis. As shown in Scheme 7A, the oxygen on the amidoxime is negatively ionized and nucleophilic to attack the carbonyl carbon of the ester to form the C–O bond, then 1,2,4-oxadiazole was constructed after the electron was transferred, -OR^3^ was cleaved, and a molecule of water was removed. As shown in Scheme 7B, using high-resistant organic base 2,4,6-trimethylpyridine as the catalyst, compound **86** was obtained by the reaction process of dehydration and cyclization from **6** and diethyl oxalate, and then 3,3’-bis (3-nitro-1,2,5-oxadiazol-4-yl)-5,5’-bi-1,2,4-oxadiazole **87** was synthetized by Tsyshevsky et al. based on nitration of **86** (Tsyshevsky et al., 2017). LLM-200 showed a theoretical density of 1.94 g cm^−3^, a detonation velocity of 8,780 m s^−1^, and a characteristic drop height (H_50_) of 62 cm, making it a high-energy, insensitive, heat-resistant explosive with good stability and detonation performance equivalent to that of RDX.

Anhydride was a strong acylating agent, which easily triggered O-acylation with amidoxime catalyzed under acidic or alkaline conditions. As the mechanism shown in Scheme 7C, anhydride was converted to carboxylic acid and carbonyl carbonium ion in acidic conditions, and then C–O bonds were formed by lone pair electrons on amidoxime attacking carbonium ions. The corresponding azo products (**89**, **91**) were obtained by Qu et al. from **6** *via* O-acylation of acetic anhydride or trifluoroacetic anhydride, cyclization, and oxidation of the KMnO_4_ / HCl system (Qu et al., 2016). (Scheme 7D)

**SCHEME 8:**
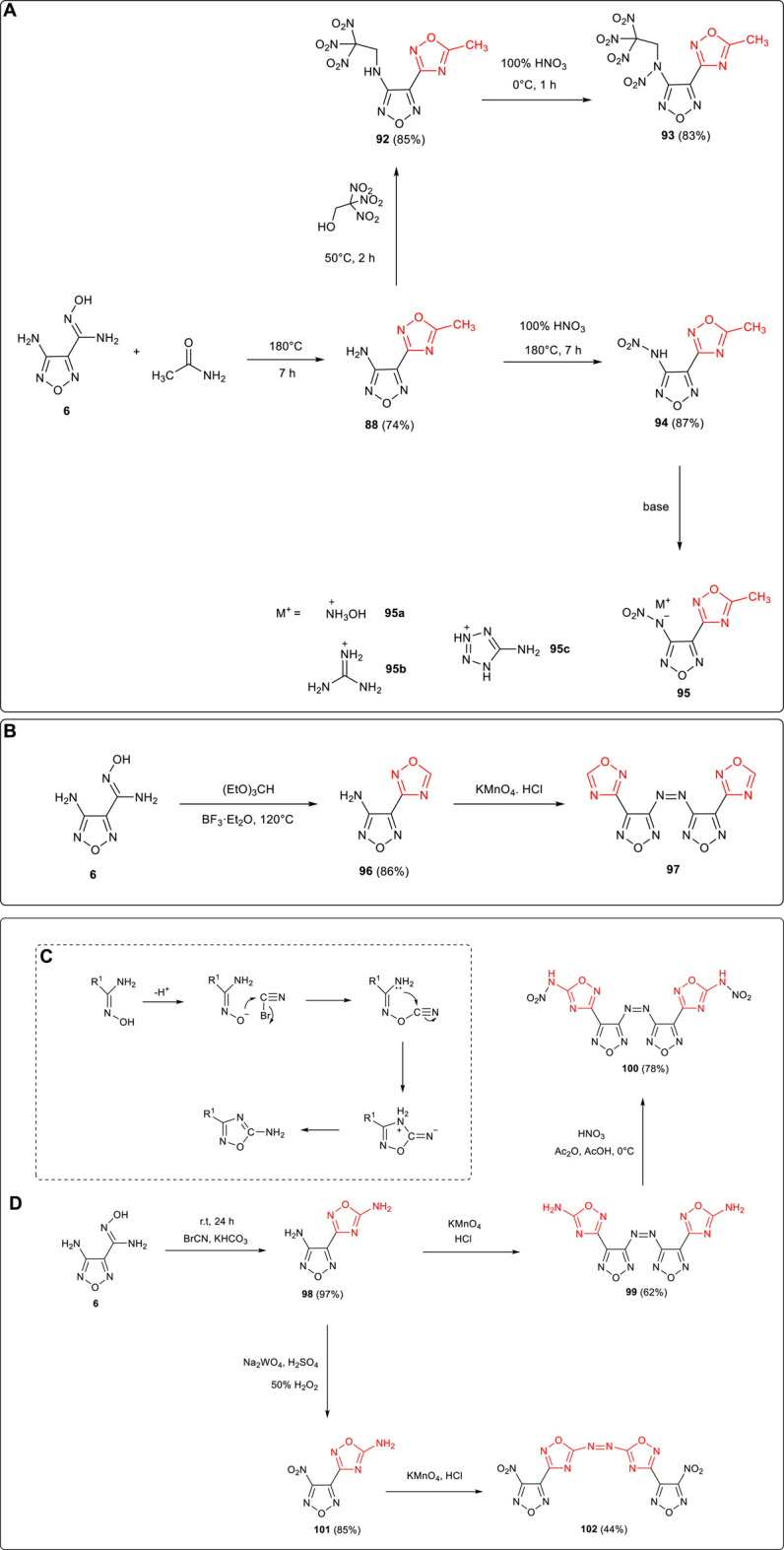
**(A)** Synthesis of **93**, **95** and its energetic ionic salts; **(B)** synthesis of **96**; **(C)** cyclization mechanism of 1,2,5-oxadiazole from BrCN; **(D)** synthesis of **100** and **102** .

1,2,4-Oxadiazole framework was normally formed by the condensation and cyclization of amide and amidoxime. 4-amino-3-(5-methyl-1,2,4-oxadiazol-3-yl)furazan **88** was synthesized by Yang et al. using **6** and acetamide as starting materials, and the active amino group on furazan was further applied in the following derivatizations (Yu et al., 2017): 1) N-(4-(5-methyl-1,2,4-oxadiazol-3-yl)-1,2,5-oxadiazol-3-yl)-N-(2,2,2-trinitroethyl) nitramide **93** (ρ: 1.78 g cm^−3^, D: 8,602 m s^−1^, P: 32.8 GPa) was obtained through the Mannich reaction of trinitroethanol catalyzed by Lewis acid which introduced the trinitroethyl energetic group, and the N-NO_2_ moiety was formed after further nitration. 2) The nitramino group was formed by the nitration of **88**, and several energetic ionic salts were obtained based on the neutralization nitramino moiety. Among them, hydroxylaminium salt **95a** exhibited good thermal stability (T_d_: 212°C) and detonation performance (D: 8,350 m s^−1^, P: 27.3 GPa) better than that of TNT (Scheme 8A). In addition, Qu et al. reported that under the catalysis of ether boron trifluoride, 4-(1,2,4-oxadiazol-3-yl)-1,2,5-oxadiazol-3-amine 96 was synthetized from 6 and (EtO)_3_CH. The azo moiety could also be formed by the oxidative coupling of 96. (Qu et al., 2016). (Scheme 8B)

Using BrCN and amidoxime as starting materials under alkaline conditions, O-acylation could be carried out and then 1,2,4-oxadiazole could be formed by dehydration and cyclization (Scheme 8C). As shown in Scheme 8D, 3-amino-4-(5-amino-1,2,4-oxadiazol-3-yl) furazan **98** was synthesized by Shaposhnikov et al. by the reaction of **6** and BrCN (Shaposhnikov et al., 2002). The activity of the amino group on furazan in **6** was higher than that of 1,2,4-oxadiazole, and **100** (ρ: 2.12 g cm^−3^, D: 10114 m s^−1^) could be obtained by the process of coupling and nitration (Qu et al., 2016). **102** (ρ: 1.92 g cm^−3^, D: 9,240 m s^−1^) could also be obtained by different reaction processes of nitration and coupling (Wang et al., 2018).

**SCHEME 9:**
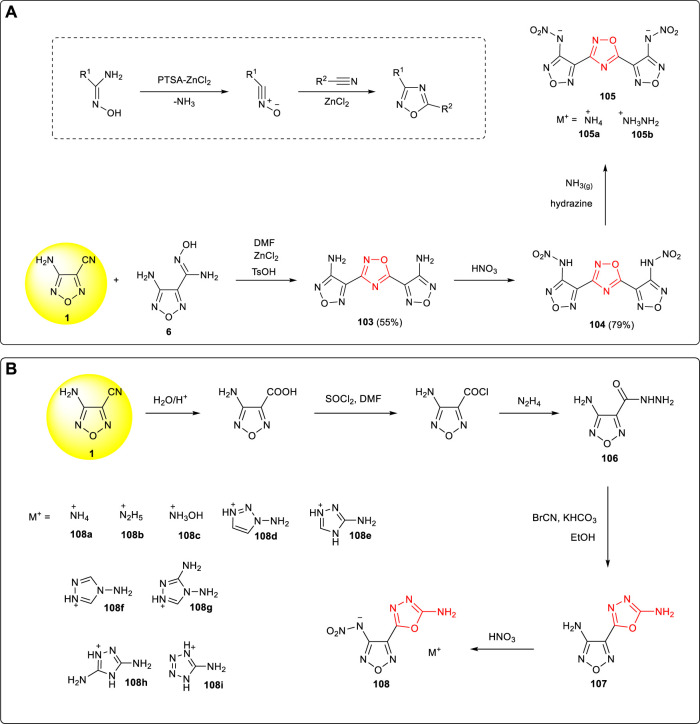
**(A)** Cyclization mechanism of 1,2,5-oxadiazole from organic nitrile and syntheses of **104** and its energetic ionic salts; **(B)** synthesis of energetic ionic salt **108a**-**108i**.

Nitrile oxide was formed after the extrusion of NH_3_ in amidoxime activated by PTSA-ZnCl_2_, and 1,2,4-oxadiazole could then be obtained by 1,3-dipole cycloaddition of nitrile oxide and organic nitrile. 3,5-bis(3-amino-furazan-4-yl)-1,2,4-oxadiazole **103** was reported by Shreeve et al. starting from the cyclization reaction of 3-amino-4-cyanofurazan **1** and the amidoxime intermediate **6** in the TsOH/ZnCl_2_ catalytic system. The nitroamino compound **104** and its energetic ionic salts were obtained by nitration and neutralization, among which the density of ammonium salt **105a** was 1.71 g cm^−3^ and the detonation velocity was 8,603 m s^−1^ (Wei et al., 2015b). (Scheme 9A)

The hydrazide intermediate **106** was prepared by the hydrolysis, acylation, and hydrazinolysis from 3-amino-4-cyanofurazan. The 3-amino-4-(2-amino-1,3,4-oxadiazol-5-yl)-1,2,5-oxadiazole **107** was then obtained by the condensation of **106** and BrCN (Tang et al., 2015). Since the amino group of 1,3,4-oxadiazole was inactive, only mononitration products could be obtained by the nitration of **107**. A series of energetic ionic salts **(108a-108i**) were synthesized by further neutralization reaction with a density of 1.61–1.81 g cm^−3^ and a theoretical detonation velocity of 7,493–8,711 m s ^−1^ (Scheme 9B).

### Hybrid Furoxan-Isoxazole–Based Energetic Compounds

**SCHEME 10:**
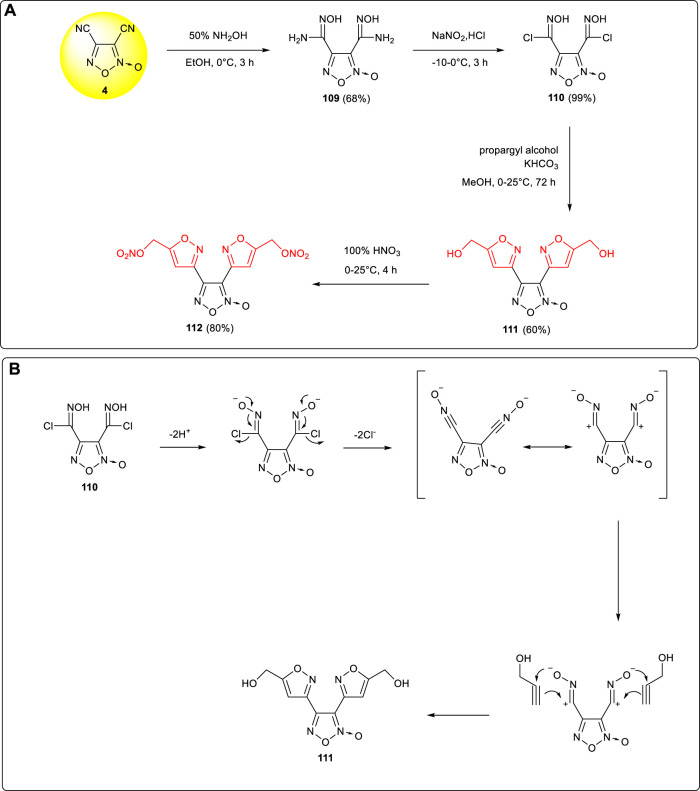
**(A)** Synthesis of **112; (B)** cyclization mechanism of isoxazole.

Wingard et al., (2016; Wingard et al., (2017) show that energetic compounds with heterocyclic framework and alkyl nitrate groups had good wetting and plasticizing properties. Compared with tetrazole or triazole, isoxazole was easier to introduce the alkyl nitrate side chain into the N-heterocyclic skeleton. 3,4-bis(5-nitroxymethylisoxazol-3-yl) furoxan **112** (ρ:1.712 g cm^−3^, m.p.:89.8°C, D:7,374 m s^−1^), which could be used as a new type of molten explosive, was synthesized by Johnson et al. from 3,4-dicyanofuroxan **4** through cyano-addition, diazotization, cyclization, and nitration (Johnson et al., 2020). (Scheme 10A) The specific cyclization mechanism is shown in Scheme 10B: a metastable coordination –C≡N→O moiety was formed from the chlorooxime group under weak base conditions, in which –C≡N→O and –C^+^ = NO^−^ are resonance forms reacted with the alkyne moiety to give an isoxazole ring structure through [3 + 2] cycloaddition.The melting point and the initial decomposition temperature of **112** are 89.8 and 193.8°C, respectively. With the detonation performance obviously better than that of TNT and low sensitivity to impact and friction (D: 8,350 m s^−1^, P: 27.3 GPa, IS: 7.8 J, FS: 240 N), **112** was regarded as a potential substitute for TNT in the formulation of melt-cast explosive.

### Fused Framework–Based Energetic Compounds

Due to the unique planar structure of fused rings, delocalization resonance of π electrons, and easier conjugate stacking effect, these compounds showed low mechanical sensitivity and high thermal stability (Zhang et al., 2018). At present, the development of fused ring energetic compounds had well-supplemented and expanded the research scope of high-energy density energetic materials.

**SCHEME 11:**
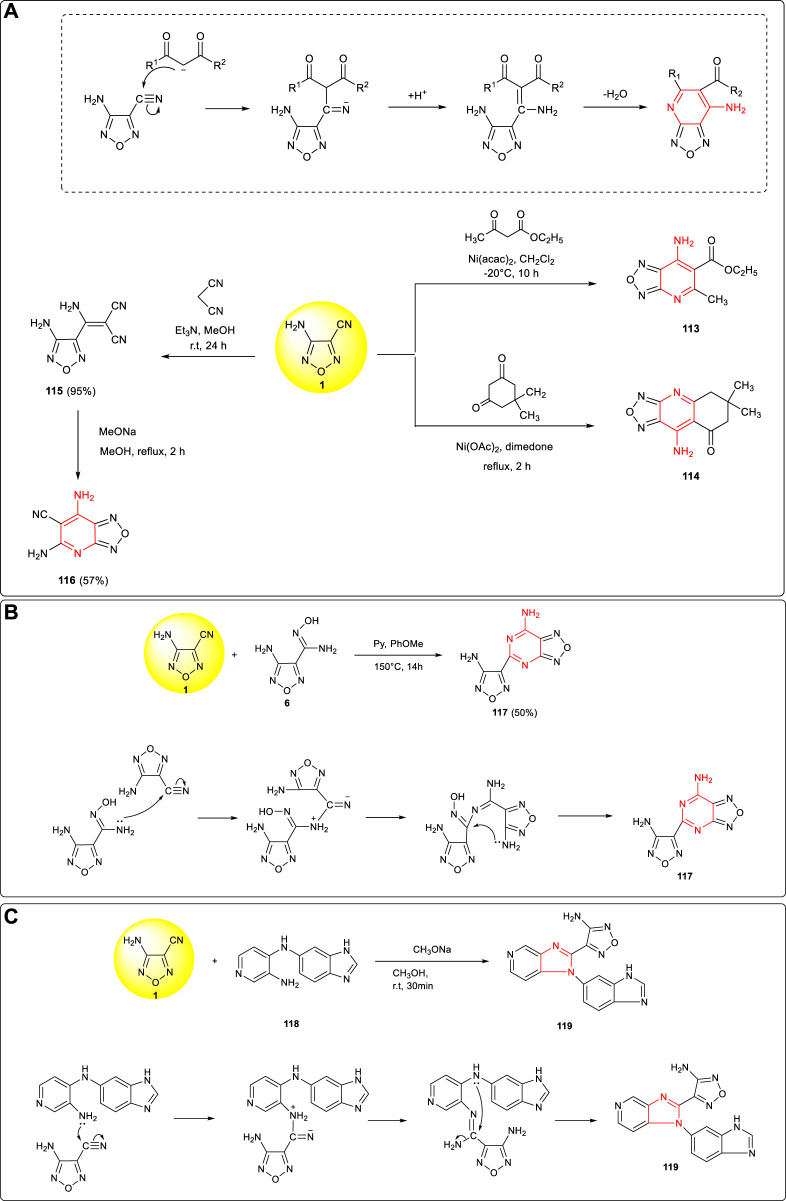
**(A)** Cyclization mechanism of pyridofurazan and synthesis of pyridofurazan intermediates 113, 114, and 116; **(B)** synthesis of 117 and cyclization mechanism of the furazanopyrimidine framework; **(C)** synthesis of 119 and cyclization mechanism of the triazolofurazan framework.

Based on the reactions between the amino group (-NH_2_) and cyano group (-CN) in 3-amino-4-cyanofurazan, a series of furazanopyridine derivatives could be obtained from the reaction of β-dicarbonyl compounds. As shown in Scheme 11A, under alkali catalysis, α-carbon anion attacked cyano carbon to form C–C bond and then the amino group attacked the carbonyl group, forming a fused ring structure after the extrusion of H_2_O. Accordingly, using 3-amino-4-cyanofurazan **1** as the starting material to react with ethyl 3-oxobutanoate and 5,5-dimethylcyclohexane-1,3-dione, the corresponding pyrido furazan fused ring compounds (**113, 114**) could be yielded (Vasil et al., 2001). Similarly, using malononitrile as the starting material to react with 3-amino-4-cyanofurazan **1**, the cyano group could be introduced into the fused ring system and 2,4-diamino-3- cyanopyridofurazan **116**, which was conducive to further energetic derivatization, was obtained by Strizhenko et al. (Strizhenko et al., 2020).

Taking 3-amino-4-cyanofurazan **1** and its amidoxime derivative **6** as the starting material, the fused ring structure furazanopyrimidine product **117** was synthetized by Pagoria et al. through high-temperature cyclization (Pagoria et al., 2017). The mechanism is shown in Scheme 11B: the lone pair electrons of the amino group (-NH_2_) in **6** attacked the carbon on the cyano group of **1** to form C–N bond, and then the lone pair electrons of the amino group attacked the = N–OH moiety to remove a molecule of NH_2_OH to form a pyrimidine ring structure. A new conjugated large π fused ring compound **119** was synthesized Vydzhak et al. using **1** and **118** (Vydzhak et al., 2020). As shown in Scheme 11C, the primary amino group in **118** attacked the cyano group, and then the lone pair electrons of the secondary amino group attacked the carbon on the cyano group to form C–N bond and eliminated a molecule of NH_3_ to form a fused ring structure. These novel cyclization reactions were valuable for further designing and synthesis of new energetic materials.

**SCHEME 12:**
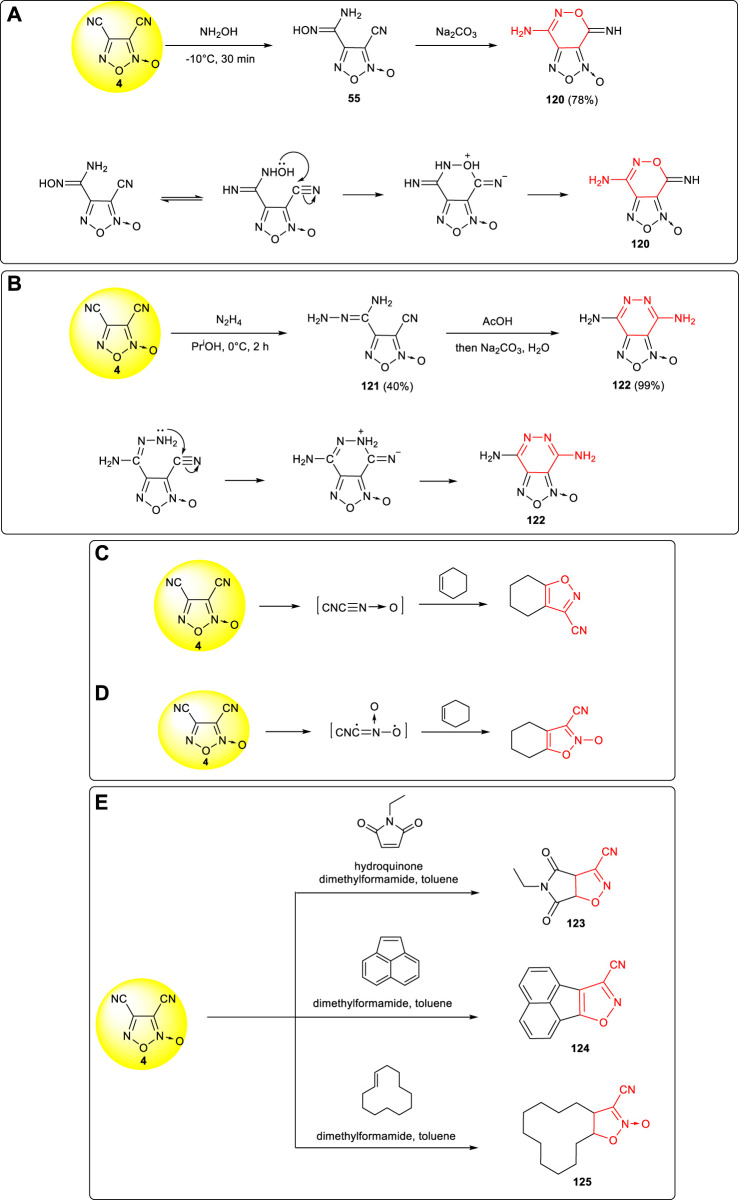
**(A)** Synthesis of 120 and its cyclization mechanism; **(B)** synthesis of 122 and the cyclization mechanism of pyridazinofurazan; **(C,D)** two cyclization mechanism of fused ring structures; **(E)** three cyano-substituted fused ring structures.

Due to the different reaction activities of the two cyano groups in 3,4-dicyanofuroxan **4**, the addition reaction usually occurred at the more reactive cyano group in position 4, and then the cyclization reaction was conducted to obtain the fused ring system. Amidoxime intermediate **55** was obtained *via* nucleophilic addition of **4** with hydroxylamine, and after the tautomerism of **55**, the hydroxyl nucleophilic attacked the carbon of the cyano group at position 3 to complete ring closure, and the fused ring compound **120** was obtained (Boyer and Pillai, 1982). (Scheme 12A) Using **4** and hydrazine as starting materials, furazanopyridine products **122** was obtained by Khisamutdinov et al. through two-step nucleophilic addition reaction (Khisamutdinov et al., 1995). As shown in Scheme 12B, the cyano group at position 3 in the cyclization process was more likely to react with the amino group on the hydrazone to form a stable six-membered ring structure.

A fused aromatic structure was formed by the 1,3-dipole cycloaddition between 3,4-dicyanofurazan and cyclic compounds containing double bonds. Two possible reaction mechanisms were involved: the first one is the formations of two molecules of cyanide through reductions (Scheme 12C), while the second one is based on the ring opening cracking which eliminates a molecule of acetonitrile to form cyanide (Scheme 12D). A variety of fused ring compounds (**123–125**) could be further designed and synthesized accordingly, and energetic groups such as tetrazolyl, hydroxytetrazolyl, dinitromethyl, and fluorodinitromethyl could be introduced to further improve the detonation performances of fused aromatic structures (Shimizu et al., 1985). (Scheme 12E)

## Conclusion

Cyano-substituted furazan and furoxan have been proven to be important intermediates for the developments of nitrogen-rich energetic compounds. In recent years, an enormous number of synthetic strategies toward energetic structures related with furazan/furoxan have been achieved based on the cyanofurazans/furoxans, revealing that these synthetic intermediates are still full of opportunities and of great interest to the chemical and material scientists around the world. This account summarized the current synthetic methods of cyanofurazan/furoxan structures, including 3-amino-4-cyanofurazan, 4-amino-3-cyano furoxan, 3,4-dicyanofurazan, and 3,4-dicyanofuroxan. Both the advantages and disadvantages of these synthetic methods were analyzed and compared. Based on the reaction activities of the amino and cyano groups in the cyanofurazan/furoxan, the synthetic strategies toward seven kinds of nitrogen-rich energetic compounds, such as azo (azoxy)-bridged, ether-bridged, methylene-bridged, hybrid furazan/furoxan-tetrazole–based, tandem furoxan–based, hybrid furazan-isofurazan–based, hybrid furoxan-isoxazole–based, and fused framework–based energetic compounds were fully reviewed. Amino groups were normally transformed into azo/azoxy or nitro moieties through oxidative processes or reacted with electrophilic structures through substitution or condensation reactions. Cyano groups showed significant advantages in cyclization processes, leading to the formations of various aromatic heterocycles such as tetrazole, furoxan, isofurazan, isoxazole, and complicated fused frameworks and the mechanisms of framework constructions were also highlighted. Furazan and furoxan structures will certainly continue to trigger increasing research in the future. With the in-depth applications of these cyanofurazan/furoxan intermediates, we believe more advanced energetic materials with excellent detonation performances, high thermal stabilities, good insensitivities to impacts/frictions, and convenient synthesis approaches will be achieved.
